# Emerging Pharmacological Strategies for Cardiac Amyloidosis: A Qualitative Analysis of Interventional Clinical Trials Registered on ClinicalTrials.Gov

**DOI:** 10.3390/jcm15041499

**Published:** 2026-02-14

**Authors:** Maan H. Harbi, Yahya A. Alzahrani

**Affiliations:** 1 Pharmacology and Toxicology Department, College of Pharmacy, Umm Al-Qura University, Makkah 21955, Saudi Arabia; 2 Department of Pharmacology, Faculty of Medicine, King Abdulaziz University, Rabigh 25732, Saudi Arabia; yaaalzahrani1@kau.edu.sa

**Keywords:** cardiac amyloidosis, transthyretin amyloid cardiomyopathy, light-chain amyloidosis, protein misfolding

## Abstract

**Introduction:** Cardiac amyloidosis, primarily comprising transthyretin amyloid cardiomyopathy (ATTR-CM) and light-chain amyloidosis with cardiac involvement (AL-cardiac), is an increasingly recognized contributor to the global heart failure burden. Management has shifted from supportive care to disease-modifying agents targeting specific stages of the amyloid cascade. This registry-based review qualitatively characterizes the current pharmacological clinical trial landscape through a registry-based analysis. **Methods:** A structured qualitative analysis of ClinicalTrials.gov was conducted for interventional trials registered between January 2015 and November 2025. Following PRISMA principles, studies were screened to include pharmacological interventions with explicit cardiac targeting while excluding neuropathy-dominant amyloidosis. Trial-level data regarding therapeutic classes, study phases, enrollment, and primary outcome domains were extracted and synthesized. **Results:** A total of 18 trials met the inclusion criteria (14 ATTR-CM; 4 AL-cardiac), representing a total enrollment of 4924 participants across 11 unique agents. Five therapeutic classes were identified: amyloid-clearing monoclonal antibodies (44.4% of trials), TTR silencers, TTR stabilizers, fibril-modifying agents, and cardiac phenotype-directed therapies. Monoclonal antibodies represented the largest class by both trial count and enrollment (3075 participants). Clinical events (n = 7) and safety/tolerability (n = 5) were the most frequent primary outcome domains. ATTR-CM trials dominated the landscape, accounting for 77.7% of the total study population, while parallel-group placebo-controlled designs were the most common study architecture (n = 10). **Conclusions:** The therapeutic landscape for cardiac amyloidosis is transitioning toward stage-specific, mechanism-based interventions. While ATTR-CM currently dominates research efforts, the expansion of silencers and monoclonal antibodies reflects an increasing capacity to intercept the amyloid cascade at distinct molecular checkpoints. However, significant heterogeneity in outcome measures and the shift toward diagnosing milder disease pose challenges for demonstrating clinical efficacy. Future priorities include standardized progression markers and addressing barriers to global access for these high-cost therapies.

## 1. Introduction

In the past, cardiac amyloidosis was regarded as a rare and atypical diagnosis. It is now recognized as an important contributor to the global burden of restrictive cardiomyopathy and heart failure [1]. Reported prevalence and mortality have increased substantially over the past two decades; as a result of improved detection and the widespread adoption of noninvasive diagnostic modalities, including bone scintigraphy and cardiac magnetic resonance imaging, rather than a true rise in disease incidence [2,3]. Earlier and more accurate diagnosis has consequently altered the clinical spectrum of cardiac amyloidosis, enabling identification of patients at less advanced stages of disease compared with historical cohorts [3,4].

Transthyretin amyloid cardiomyopathy (ATTR-CM) and light-chain amyloidosis with cardiac involvement (AL-cardiac) are the two main types of cardiac amyloidosis. They have different pathophysiological causes and clinical courses. ATTR-CM is caused by misfolding of transthyretin, a transport protein produced by the liver, into insoluble fibrils that accumulate within the myocardium, producing a slowly progressive infiltrative cardiomyopathy that predominantly affects older adults [5]. In contrast, AL-cardiac arises from a plasma cell dyscrasia characterized by overproduction of monoclonal immunoglobulin light chains, which exert direct cardiotoxic effects in addition to forming myocardial deposits [6].

Although transthyretin amyloid cardiomyopathy (ATTR-CM) has gained increasing recognition in recent years, light-chain amyloidosis (AL) continues to account for a substantial proportion of cardiac amyloidosis cases in routine clinical practice [7]. Epidemiological studies indicate that AL amyloidosis frequently predominates among patients with cardiac involvement, particularly in referral-based cohorts [8]. In contrast, the interventional clinical trial landscape is largely focused on ATTR-CM, reflecting the greater number of tractable therapeutic targets within the transthyretin pathway and the feasibility of organ-specific disease-modifying strategies [9]. This mismatch between real-world disease burden and therapeutic development highlights the importance of interpreting emerging pharmacological advances within both biological and clinical contexts.

In the past, the only management of cardiac amyloidosis available was supportive heart failure therapy and symptom control [5]. At that time, disease-modifying (amyloid-directed) pharmacological options were limited. Progress in understanding the molecular and cellular mechanisms underlying amyloid formation led to the development of pharmacological strategies that target multiple stages of the amyloid cascade [10]. Contemporary approaches include transthyretin stabilizers that inhibit tetramer dissociation, gene-silencing therapies that reduce hepatic production of amyloidogenic precursor proteins, and amyloid-clearing monoclonal antibodies designed to promote removal of established myocardial deposits [11]. The emergence of these mechanism-based therapies has resulted in a rapidly expanding and increasingly heterogeneous clinical trial landscape, underscoring the need for structured synthesis of ongoing pharmacological development efforts.

In rare and evolving disease areas such as cardiac amyloidosis, registry-based analyses play a critical role in contextualizing therapeutic innovation and identifying research trends that may not yet be reflected in the published literature [12,13,14]. The ClinicalTrials.gov registry provides a comprehensive and standardized platform for capturing global interventional activity, facilitating evaluation of trial design, therapeutic mechanisms, and outcome priorities as new pharmacological strategies emerge.

Accordingly, the aim of this review is to perform a qualitative, registry-based analysis of interventional clinical trials registered on ClinicalTrials.gov to characterize current pharmacological strategies for cardiac amyloidosis, with particular emphasis on therapeutic drug classes, primary outcome domains, and the frequency of investigational agents.

## 2. Materials and Methods

This review was conducted as a qualitative, registry-based analysis of interventional clinical trials evaluating pharmacological treatment strategies for cardiac amyloidosis. The screening and selection process was adapted from the PRISMA statement to ensure transparent reporting of study identification, screening, eligibility assessment, and inclusion. The objective was to synthesize trial-level evidence describing emerging and established pharmacological approaches relevant to cardiac amyloidosis without performing quantitative meta-analysis or formal risk-of-bias assessment, consistent with a descriptive registry analysis.

Clinical trial data were obtained exclusively from the ClinicalTrials.gov registry, a publicly accessible database maintained by the U.S. National Library of Medicine. This registry was selected as the sole data source due to its comprehensive coverage of global interventional trials, standardized reporting structure, and suitability for comprehensive identification and qualitative synthesis of ongoing and completed pharmacological studies.

A structured search of ClinicalTrials.gov was performed using predefined condition-related terms relevant to cardiac amyloidosis. The initial search combined the terms “cardiac amyloidosis”, “ATTR cardiomyopathy”, “transthyretin cardiomyopathy,” and “AL amyloidosis.” The search was restricted to interventional studies involving adult participants, including both adults aged 18–64 years and older adults aged 65 years or above. Eligible study phases included Early Phase 1, Phase 1, Phase 2, Phase 3, Phase 4, and studies categorized as not applicable. Trials with recruitment statuses of not yet recruiting, recruiting, active but not recruiting, completed, enrolling by invitation, or unknown status were included. The search was limited to trials first posted between 1 January 2015 and 30 November 2025. This initial search yielded 187 records.

To ensure the analysis remained specific to the cardiac phenotype, neuropathy-dominant forms of transthyretin amyloidosis were excluded. Records containing the terms neuropathy, polyneuropathy, FAP, or familial amyloid polyneuropathy were removed unless the trial explicitly targeted a mixed population with primary cardiac endpoints. Application of these exclusion criteria reduced the dataset to 102 records, which were subjected to manual screening.

The remaining records were screened by reviewing study titles, condition descriptions, intervention types, and brief summaries to assess relevance to pharmacological treatment of cardiac amyloidosis. Studies were excluded at this stage if they were observational, diagnostic, imaging-only, device-based, procedural, or focused solely on supportive or symptomatic management. Additional exclusions included pediatric studies, trials targeting localized or non-systemic amyloidosis, systemic light-chain amyloidosis trials without explicit cardiac relevance, and studies lacking sufficient methodological information for qualitative assessment. To ensure the analysis focused on the management of cardiomyopathy, trials targeting systemic light-chain (AL) amyloidosis were subject to specific inclusion criteria regarding cardiac relevance. Studies were classified as ‘AL-cardiac’ only if they demonstrated explicit cardiac targeting through: (1) inclusion criteria restricted to patients with cardiac involvement, specifically those defined by Mayo Stage categories (e.g., Stage IIIa/b) which rely on cardiac biomarkers (troponin and NT-proBNP) or (2) the utilization of primary endpoints specific to heart failure morbidity, such as cardiovascular-related hospitalizations or the Kansas City Cardiomyopathy Questionnaire (KCCQ). Systemic AL amyloidosis trials focusing solely on hematologic response to chemotherapy without explicit cardiac selection or organ-specific outcomes were excluded from this review.

Given the significant heterogeneity regarding study phases, sample sizes, and outcome definitions across the included trials, a descriptive qualitative synthesis was performed rather than a quantitative meta-analysis. To address factors influencing trial interpretability, data were stratified by therapeutic class and amyloidosis subtype (ATTR-CM vs. AL-cardiac). Factors potentially influencing results, such as the variability in primary outcome domains (e.g., hard clinical events versus surrogate biomarkers) and differences in trial design (e.g., placebo-controlled versus open-label), were explicitly extracted and categorized to characterize the maturity of the evidence base for each drug class.

**Figure 1:**
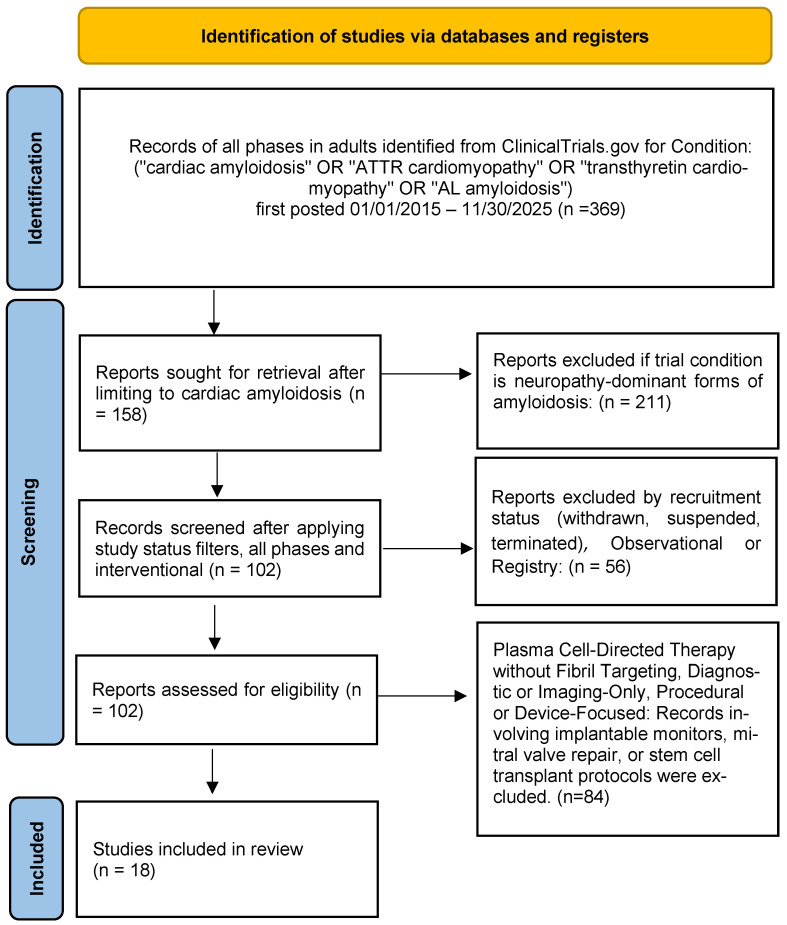
PRISMA flow diagram summarizing the identification and selection of interventional clinical trials of pharmacological therapies for cardiac amyloidosis from ClinicalTrials.gov.

Full registry entries of potentially eligible trials were then reviewed in detail to confirm cardiac disease relevance, pharmacological intent, and eligibility. Following this screening and eligibility assessment process, 18 interventional clinical trials were included in the final qualitative synthesis (Figure 1).

For each included study, relevant trial-level data were extracted from ClinicalTrials.gov and recorded in a structured spreadsheet. Extracted information included the trial registration number, study title, amyloidosis subtype, investigational drug or biological agent, therapeutic mechanism of action, study phase, recruitment status, planned or actual enrolment, primary outcome domain, and study design with comparator characteristics. Therapeutic class assignment was based on the dominant mechanism of action of the investigational intervention, allowing classification into transthyretin stabilizers, transthyretin silencers, amyloid-clearing monoclonal antibodies, amyloid disruptors or fibril-modifying agents, and cardiac phenotype-directed therapies relevant to transthyretin amyloid cardiomyopathy.

**Figure 2:**
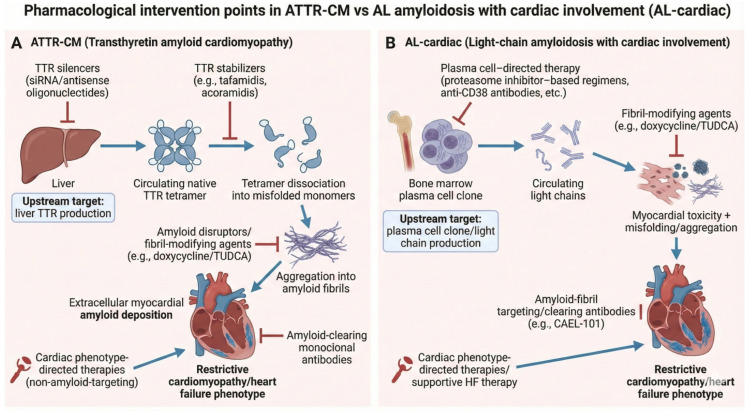
Pharmacological intervention points in ATTR-CM versus AL amyloidosis with cardiac involvement. Comparison of the amyloid cascades and therapeutic targets in transthyretin amyloid cardiomyopathy (ATTR-CM) and light-chain amyloidosis with cardiac involvement (AL-cardiac). In ATTR-CM, liver-derived transthyretin misfolds following tetramer dissociation, aggregates into amyloid fibrils, and deposits within the myocardium, leading to restrictive cardiomyopathy. Therapeutic strategies target distinct stages of this cascade, including TTR silencers, TTR stabilizers, fibril-modifying agents, amyloid-clearing monoclonal antibodies, and downstream phenotype-directed cardiac therapies. In AL-cardiac disease, a bone marrow plasma cell clone produces toxic monoclonal light chains that drive myocardial injury and amyloid deposition; upstream plasma cell-directed therapy is the cornerstone of treatment, with fibril-targeting strategies and supportive cardiac care serving adjunctive roles. (Arrows refer to pathophysiology, Perpendicular Lines refer to site of drug action).

A descriptive qualitative synthesis was performed to summarize the characteristics of the included trials according to therapeutic class, outcome domains, and study design features. Given the heterogeneity of pharmacological interventions, trial designs, and outcome measures, no quantitative synthesis or meta-analysis was undertaken. Given the registry-based nature of the data source, no patient-level variables or confounding factors were analyzed, no inferential statistical analyses were performed, and ethical approval and informed consent were not required. Gemini AI was used to generate graphical figure of amyloid cascade and relevant therapeutic targets (Figure 2).

## 3. Results

### 3.1. Study Selection

After initial registry filtering, structured screening of the ClinicalTrials.gov dataset yielded 102 records for review. Following application of predefined inclusion and exclusion criteria focused on interventional pharmacological trials with explicit cardiac targeting or disease-modifying (amyloid-directed) intent, 18 trials were selected for inclusion in this review.

### 3.2. General Characteristics of Included Trials

The 18 included trials primarily targeted two disease subtypes: ATTR-CM and AL-cardiac. As detailed in Table 1**,** fourteen trials focused on ATTR-CM, while four trials enrolled patients with AL-cardiac disease. The studies spanned clinical phases 1 through 4, with phase 3 trials representing the most common design. Planned or actual enrolment varied widely, ranging from small exploratory studies enrolling approximately 15 participants to large phase 3 trials enrolling up to 1400 participants (Table 1). Enrolment was not reported for one included trial.

Four trials investigating therapies for AL amyloidosis were classified as the ‘AL-cardiac’ subtype (Table 1). Although titled under the broader disease heading, these specific studies were distinguished by their restriction to populations with advanced cardiac involvement, such as patients with Mayo Stage IIIa or IIIb disease, For example, the CAEL-101 program (NCT04512235, NCT04504825) prioritized cardiac-specific primary endpoints, including overall survival, cardiovascular-related hospitalizations, and functional status (KCCQ), rather than standard hematologic remission alone. Similarly, the doxycycline trial (NCT03401372) was included based on its specific rationale to improve survival in cardiac-staged AL patients through fibril disruption. Consequently, these trials represent a distinct subset of AL research focused on improving organ function via fibril-targeting or disrupting mechanisms, as opposed to solely targeting the plasma cell clone.

### 3.3. Pharmacological Strategies Evaluated

Five distinct therapeutic classes were identified among the pharmacological strategies evaluated. These included transthyretin (TTR) stabilizers, TTR silencers (small interfering RNA or antisense oligonucleotides), amyloid-clearing monoclonal antibodies, amyloid disruptors or fibril-modifying agents, and cardiac phenotype-directed therapies in ATTR-CM. These agents (e.g., trimetazidine, empagliflozin) were classified separately as concomitant or supportive interventions, as they target metabolic or hemodynamic pathways rather than the underlying amyloidogenic process. Classification was based on the dominant mechanism of action of each investigational intervention, as summarized in Table 1.

### 3.4. Frequency of Investigational Agents

The distribution of individual investigational agents across the included trials is presented in Table 2. CAEL-101 and eplontersen were the most frequently evaluated agents, each studied in three trials. ALXN2220, AG10/ALXN2060, and doxycycline were each evaluated in two trials. Other agents including NI006, coramitug, AT-02, tafamidis, trimetazidine, and empagliflozin were each assessed in a single interventional study.

### 3.5. Distribution of Therapeutic Drug Classes

As shown in Table 3, amyloid-clearing monoclonal antibodies constituted the largest therapeutic class, accounting for 8 of the 18 trials (44.4%) and the highest total reported enrolment (3075 participants). This class included four ATTR-CM trials and four AL-cardiac trials. TTR silencers (n = 3) and TTR stabilizers (n = 3) followed, with total reported enrolments of 1481 and 127 participants, respectively. Amyloid disruptors or fibril-modifying agents were evaluated in two trials enrolling 202 participants, while cardiac phenotype-directed supportive (adjunct) therapies were assessed in two trials with a combined enrolment of 39 participants.

### 3.6. Primary Outcome Domains

Primary endpoints across the included trials were categorized into six outcome domains, as summarized in Table 4. Clinical events including all-cause mortality and hospitalization were the most frequently reported primary outcome domain, used in seven trials. Safety and tolerability were the primary endpoints in five trials, including the safety-focused CAEL-101 study. Biomarker or pharmacodynamic endpoints, such as transthyretin levels or stabilization, were primary outcomes in three trials. Cardiac structural or functional assessment using imaging modalities (e.g., echocardiography, cardiac magnetic resonance) served as the primary outcome in two trials, while functional or health-status measures (six-minute walk distance) were the primary endpoint in one trial. None of the included trials used hematologic or organ response as the sole primary outcome domain.

### 3.7. Trial Design and Comparator Characteristics

Trial design and comparator characteristics are detailed in Table 5. Parallel-group placebo-controlled designs were the most common, utilized in ten trials. Six trials employed a single-arm, open-label design without a reported comparator. Parallel-group active-controlled designs were used in two trials, both comparing investigational therapy with standard of care. One trial utilized a crossover design with a placebo comparator.

### 3.8. Summary of Results

In summary, 18 interventional clinical trials met the eligibility criteria for this review, representing a total reported enrolment of 4924 participants across 11 unique pharmacological agents. Amyloid-clearing monoclonal antibodies were the most prevalent therapeutic class by both trial count and enrolment volume. Clinical events and safety were the most common primary outcome domains, and parallel-group placebo-controlled designs predominated. The distribution of included trials was weighted toward ATTR-CM, which accounted for 77.7% of the study population.

## 4. Discussion

The structured search across the clinical trials registry identified therapeutic strategies targeting different stages of the amyloid cascade. Transthyretin (TTR) silencers act by degrading messenger RNA (mRNA) and thereby reducing hepatic synthesis of the amyloid precursor protein [1]. TTR stabilizers act by slowing the formation of new amyloid deposits through binding to the natural tetramer preventing its breakdown to monomers [15]. In contrast, amyloid-clearing monoclonal antibodies are designed to recognize epitopes on deposited fibrils, promoting immune-mediated clearance of established myocardial amyloid [16]. Fibril-modifying agents act by interfering with fibril assembly or altering fibril structure to enhance their clearance [17]. Cardiac phenotype-directed therapies represent a distinct category, since they do not directly target amyloid pathology but focus on myocardial function and the alleviation of heart failure symptoms within the framework of infiltrative cardiomyopathy [18].

Although AL-cardiac amyloidosis is clinically more prevalent, clinical trial representation shows an imbalance favoring ATTR-CM. This divergence reflects fundamental differences in disease biology and clinical progression [19]. ATTR-CM typically follows a slowly progressive course over many years, creating a relatively stable platform for long-duration cardiovascular trials centered on clinical outcomes [15]. AL-cardiac amyloidosis, on the other hand, is often an aggressive disease associated with high early mortality. In contrast, AL-cardiac amyloidosis often presents as an aggressive disease with high early mortality. Consequently, AL-cardiac frequently necessitates urgent hematologic intervention and limits the feasibility of placebo-controlled designs or delayed treatment initiation [20]. Furthermore, the therapeutic development for each type has taken different courses. For AL, research has been driven by hematology-led plasma cell dyscrasia trials, whereas ATTR-CM studies have followed a cardiology-focused model targeting a single, organ-specific amyloidogenic protein [21].

The diversity of primary outcomes in cardiac amyloidosis clinical trials adds some difficulty in interpreting current trials. In cases of clinical trials of rare diseases, it is challenging to follow clinical event endpoints as they require large sample sizes and prolonged follow-up [22]. Functional assessments, including six-minute walk distance and health-status questionnaires, offer sensitivity to change but may be influenced by non-cardiac factors such as age, frailty, or coexisting neuropathy [23]. Moreover, earlier diagnosis and improved disease awareness have shifted trial populations toward milder baseline disease, resulting in slower progression within placebo groups compared with historical cohorts. Consequently, detectable differences between active treatment and control arms may be less relative to earlier landmark studies [19,24].

The predominance of transthyretin amyloid cardiomyopathy (ATTR-CM) trials identified in this review is consistent with contemporary international guidelines and expert consensus documents, which position transthyretin stabilization as the current therapeutic standard while recognizing gene-silencing approaches as rapidly emerging disease-modifying options [25]. In parallel, the high frequency of amyloid-clearing monoclonal antibodies observed across registered interventional trials reflects a broader mechanistic shift described in recent expert pathways, which highlight fibril depletion as a promising strategy for patients with established myocardial amyloid burden [26].

In contrast, although international recommendations emphasize urgent plasma cell-directed therapy as the cornerstone of management for light-chain (AL) amyloidosis, the present review demonstrates a persistent underrepresentation of cardiology-focused interventional trials in AL-cardiac disease compared with ATTR-CM [22]. This imbalance has been widely attributed to the distinct clinical presentation of the two subtypes; while ATTR-CM typically follows a slowly progressive course that permits long-duration, placebo-controlled cardiovascular trials, AL amyloidosis often presents as a rapidly progressive hematologic emergency, limiting the feasibility of delayed intervention or hard cardiovascular endpoints [22].

The heterogeneity of primary outcome domains identified in this review further mirrors challenges highlighted in the recent international literature. Guidelines and expert reviews increasingly advocate for clinically meaningful endpoints that capture functional status, quality of life, and long-term outcomes; however, as disease recognition shifts toward earlier and milder phenotypes, event rates in control arms decline, reducing the sensitivity of traditional mortality-based endpoints [27]. This evolving landscape underscores the need for harmonized outcome frameworks capable of aligning trial design with contemporary guideline expectations and real-world clinical trajectories [6].

Clinical positioning of available and emerging therapies appears closely linked to disease stage along both biological and clinical progression [1,28]. In early or pre-symptomatic stages, limiting amyloid formation by upstream precursor suppression (TTR silencers) is most relevant to prevent irreversible myocardial injury [29]. Meanwhile, TTR stabilizers and amyloid-clearing antibodies serve as foundational mid-cascade intervention in symptomatic patients or positioned as adjunctive therapies in individuals with substantial residual amyloid burden, respectively [30]. In AL-cardiac amyloidosis, rapid suppression of the plasma cell clone remains the primary therapeutic priority, with fibril-targeting agents considered potential adjuncts only after adequate hematologic control has been achieved [31,32]. Although conceptual sequencing strategies including early combination approaches have been proposed, registry data indicate that robust comparative evidence to guide such strategies remains limited [11,33].

The key unmet needs in pharmacological treatment of cardiac amyloidosis direct us to the future research focus. Although contemporary therapies attenuate disease progression, they do not provide consistent reversal of myocardial damage [34,35]. To improve care, researchers are focusing on several key areas; there is a need for highly sensitive biomarkers to define therapeutic response [36]. Multiple blood-based candidates are under evaluation in ATTR, including circulating TTR concentration, retinol-binding protein 4, and TTR aggregate-specific probes such as TAD1 [37,38]. It is critical to set standards for disease progression and for functional and quality of life measures, as consensus-driven, prospectively validated standards are needed [39,40,41]. Many of these novel disease-modifying (amyloid-directed) therapies are high-priced, which prevents many patients around the world from getting fair and equal access to treatment. Because this disease affects many parts of the body, heart specialists (cardiologists) and blood specialists (hematologists) must work together more closely. This teamwork will help design clinical trials that focus on what matters most to the patient: feeling better, staying independent, and enjoying a higher quality of life.

Reviews that are based on registry-based data have a few inherent limitations, which must be considered when interpreting these findings [14]. First, as a descriptive analysis rather than a meta-analysis of results, no formal risk-of-bias assessment was performed, and no statistical inferences regarding treatment efficacy were made. Second, relying exclusively on ClinicalTrials.gov may exclude early-phase or locally sponsored international studies not registered in this database. Public registries also suffer from variable data quality, inconsistent coding and missing entries, as we found in one of the included studies did not report enrollment numbers. There is also a real risk that these public records make the research landscape look more advanced than it actually is, as registries are not always updated to show when a trial is terminated or ends in a negative result. Significant trial heterogeneity further complicates the synthesis, particularly regarding the evolving baseline characteristics of the study populations. Newer trials like HELIOS-B and ATTRibute-CM are enrolling “healthier” participants who were diagnosed at much earlier stages than the people in older landmark studies like ATTR-ACT. This makes finding measurable differences between drug and placebo arms harder and smaller to detect. Finally, the diversity of primary outcome domains that exists between studies (Table 4), limits the ability to perform direct comparisons across different pharmacological classes. Finally, while ATTR-CM and AL-cardiac trials were analyzed in parallel to respect their biological distinctions, the aggregate presentation of landscape trends should be interpreted with caution given the heterogeneity of these conditions’ cohorts.

## 5. Conclusions

The shift in the pharmacological management of cardiac amyloidosis transitioning from purely palliative symptomatic care toward stage-specific, disease-modifying (amyloid-directed) strategies represents a fundamental change in clinical practice. Recent advances in transthyretin silencers and the development of amyloid-clearing monoclonal antibodies suggest a broader capacity to intercept the amyloid cascade at distinct molecular checkpoints. While refined diagnostic protocols now facilitate detection in earlier, milder phases of the disease, this improved prognostic outlook complicates the selection of clinical trial endpoints and the interpretation of long-term therapeutic efficacy. This situation highlights the need for robust, data-driven guidelines on which drugs to use first, how to combine them safely, and standardized protocols for monitoring a patient’s health over time.

## Figures and Tables

**Table 1:** Summary of Included Interventional Clinical Trials for Cardiac Amyloidosis. This table summarizes key trial-level characteristics, including amyloidosis subtype, clinical phase, therapeutic class, investigational agent, and planned or reported enrollment. Therapeutic classes were assigned according to the dominant biological mechanism of action of the investigational intervention. Trials enrolling mixed populations were classified based on their primary cardiac-targeted therapeutic intent.

NCT Number	Study Title	Amyloidosis Subtype (ATTR-CM or AL-Cardiac)	Intervention	Therapeutic Class	Study Phase	Enrolment (Planned or Actual)
**NCT07213583**	Study of Re-Treatment With ALXN2220 in Patients With ATTR-CM	ATTR-CM	ALXN2220	Amyloid-clearing monoclonal antibodies	Phase 2	35
**NCT04512235**	A Study to Evaluate the Efficacy and Safety of CAEL-101 in Patients With Mayo Stage IIIa AL Amyloidosis (CARES)	AL-cardiac	CAEL-101	Amyloid-clearing monoclonal antibodies	Phase 3	125
**NCT04504825**	A Study to Evaluate the Efficacy and Safety of CAEL-101 in Patients With Mayo Stage IIIb AL Amyloidosis (CARES)	AL-cardiac	CAEL-101	Amyloid-clearing monoclonal antibodies	Phase 3	284
**NCT04304144**	A Study to Evaluate the Safety and Tolerability of CAEL-101 in Patients With AL Amyloidosis	AL-cardiac	CAEL-101	Amyloid-clearing monoclonal antibodies	Phase 2	25
**NCT05633563**	The Effect of Trimetazidine on Mitochondrial Function and Myocardial Performance in ATTR-CM	ATTR-CM	Trimetazidine	Cardiac phenotype-directed supportive (adjunct) therapies (non-amyloid-targeting)	Phase 4	24
**NCT04360434**	First-in-Human Study of NI006 in Patients With ATTR-CM	ATTR-CM	NI006	Amyloid-clearing monoclonal antibodies	Phase 1	46
**NCT03481972**	Doxycycline/TUDCA Plus Standard Supportive Therapy Versus Standard Supportive Therapy Alone in Cardiac Amyloidosis caused by transthyretin	ATTR-CM	Doxycycline/TUDCA	Amyloid disruptors/fibril-modifying agents	Phase 3	102
**NCT03458130**	Study of AG10 in Amyloid Cardiomyopathy	ATTR-CM	AG10	TTR stabilizers	Phase 2	49
**NCT04843020**	ION-682884 in Patients With TTR Amyloid Cardiomyopathy	ATTR-CM	ION 682884	TTR silencers (siRNA/antisense oligonucleotides)	Phase 2	17
**NCT07207811**	CLEOPATTRA Coramitug Study in ATTR-CM	ATTR-CM	Coramitug	Amyloid-clearing monoclonal antibodies	Phase 3	1280
**NCT06194825**	EPIC-ATTR: Eplontersen in Chinese Subjects With ATTR-CM	ATTR-CM	Eplontersen	TTR silencers (siRNA/antisense oligonucleotides)	Phase 3	64
**NCT06183931**	Study of ALXN2220 Versus Placebo in Adults With ATTR-CM	ATTR-CM	ALXN2220	Amyloid-clearing monoclonal antibodies	Phase 3	1160
**NCT04814186**	Tafamidis in Chinese Participants With ATTR-CM	ATTR-CM	Tafamidis	TTR stabilizers	Phase 3	53
**NCT04622046**	A Phase 3 Study of ALXN2060 in Japanese Participants With Symptomatic ATTR-CM	ATTR-CM	ALXN2060	TTR stabilizers	Phase 3	25
**NCT04136171**	CARDIO-TTRansform: Eplontersen in Participants With ATTR-CM	ATTR-CM	Eplontersen	TTR silencers (siRNA/antisense oligonucleotides)	Phase 3	1400
**NCT03401372**	BCD With or Without Doxycycline in Mayo Stage II-III AL	AL-cardiac	Doxycycline	Amyloid disruptors/fibril-modifying agents	not reported	not reported
**NCT05233163**	SGLT2 Inhibitors in Transthyretin Amyloid Cardiomyopathy	ATTR-CM	Empagliflozin	Cardiac phenotype-directed supportive (adjunct) therapies (non-amyloid-targeting)	Phase 4	15
**NCT05951049**	A Study of AT-02 in Subjects With Systemic Amyloidosis	AL-cardiac	AT-02	Amyloid-clearing monoclonal antibodies	Phase 2	120

Abbreviations: AL, light-chain amyloidosis; AL-cardiac, light-chain amyloidosis with cardiac involvement; ATTR-CM, transthyretin amyloid cardiomyopathy; NCT, National Clinical Trial identifier; TTR, transthyretin. Therapeutic class assignment was based on the dominant mechanism of action of the investigational agent as specified in the ClinicalTrials.gov registry entry. Trials enrolling mixed amyloidosis populations were classified according to the primary cardiac-targeted intervention. Enrollment numbers reflect planned or reported enrollment at the time of registry extraction.

**Table 2:** Frequency of Pharmacological Agents Evaluated in Cardiac Amyloidosis Trials. The table reports the number of registered interventional trials in which each pharmacological agent was evaluated. Each agent was counted once per trial regardless of dosing regimen, treatment duration, or combination therapy. Extension and re-treatment studies registered as independent trials were counted separately.

Drug/Agent	Therapeutic Class	Number of Trials
CAEL-101	Amyloid-clearing monoclonal antibodies	3
Eplontersen	TTR silencers (siRNA/antisense oligonucleotides)	3
ALXN2220	Amyloid-clearing monoclonal antibodies	2
AG10 (ALXN2060)	TTR stabilizers	2
Doxycycline	Amyloid disruptors/fibril-modifying agents	2
NI006	Amyloid-clearing monoclonal antibodies	1
Coramitug	Amyloid-clearing monoclonal antibodies	1
AT-02	Amyloid-clearing monoclonal antibodies	1
Tafamidis	TTR stabilizers	1
Trimetazidine	Cardiac phenotype-directed supportive (adjunct) therapies (non-amyloid-targeting)	1
Empagliflozin	Cardiac phenotype-directed supportive (adjunct) therapies (non-amyloid-targeting)	1

**Table 3:** Distribution of Therapeutic Drug Classes in Interventional Cardiac Amyloidosis Trials. Therapeutic strategies are grouped according to their primary mechanism of action within the amyloid cascade. Cardiac phenotype-directed supportive (adjunct) therapies refer to interventions that target myocardial function or heart failure symptoms without directly modifying amyloid production, aggregation, or clearance. Ratios are calculated using the total number of included trials as the denominator.

Drug Class	Number of Trials (n/N)	Total Enrolment	ATTR-CM vs. AL-Cardiac (Counts)	Primary Outcome Focus (Brief)	Typical Design/Comparator (Brief)	Notes (1 Line Max)
TTR stabilizers	3/18	127	3 ATTR/0 AL	Biomarkers/pharmacodynamics; functional/health status	Randomized parallel; placebo	Targets protein tetramer stability to prevent amyloidogenesis.
TTR silencers (ASO/siRNA)	3/18	1481	3 ATTR/0 AL	Clinical events; biomarkers/pharmacodynamics	Randomized parallel; placebo	Evaluates reduction in hepatic TTR production via genetic silencing.
Amyloid-clearing monoclonal antibodies	8/18	3075	4 ATTR/4 AL	Clinical events; imaging; safety/tolerability	Randomized parallel; placebo	Includes safety-focused study for CAEL-101 and trials for cardiac-staged AL.
Amyloid disruptors/fibril-modifying agents	2/18	202	1 ATTR/1 AL	Clinical events	Randomized parallel; standard-of-care	Classified by doxycycline (fibril-modifying) component; background plasma cell therapy SoC.
**Cardiac phenotype-directed supportive (adjunct) therapies (non-amyloid-targeting)**	2/18	39	2 ATTR/0 AL	Functional/health status; imaging; safety/tolerability	Randomized crossover; placebo or single group	Focuses on myocardial performance and heart failure symptom management.

Abbreviations: AL, light-chain amyloidosis; AL-cardiac, light-chain amyloidosis with cardiac involvement; ATTR-CM, transthyretin amyloid cardiomyopathy; ASO, antisense oligonucleotide; siRNA, small interfering RNA; TTR, transthyretin. Therapeutic drug classes were defined according to the principal biological target within the amyloid cascade. Cardiac phenotype-directed therapies were defined as pharmacological agents that improve myocardial function or heart failure symptoms without directly targeting amyloid production, aggregation, or clearance. Percentages were calculated using the total number of included trials as the denominator.

**Table 4:** Primary Outcome Domains in Interventional Cardiac Amyloidosis Trials. Trials were categorized according to the prespecified primary endpoint reported in the ClinicalTrials.gov registry. Outcome domains include clinical events, functional or health status measures, biomarkers or pharmacodynamic endpoints, cardiac imaging or structural assessments, and safety or tolerability outcomes. Trials reporting multiple primary endpoints were classified according to the dominant outcome domain most relevant to the study objective.

Outcome Domain	Frequency (Number of Trials)	Example Outcomes/Measures (Brief)
Clinical events (mortality/hospitalization)	7	All-cause mortality, cardiovascular-related hospitalizations, and progression-free survival.
Functional/health status (6MWD/KCCQ)	2	Distance walked during the 6 min walk test (6MWT).
Biomarkers/pharmacodynamics (NT-proBNP, TTR levels)	3	Percent stabilization of TTR tetramers and reduction in serum TTR concentration.
Cardiac imaging/structure (echo/GLS, CMR/ECV/LV mass, scintigraphy)	2	Change in myocardial amyloid burden assessed by echocardiography, cardiac magnetic resonance (ECV/LV mass), or nuclear scintigraphy.
Safety/tolerability (TEAEs/SAEs)	5	Incidence and severity of treatment-emergent adverse events (TEAEs) and serious adverse events (SAEs).
Hematologic/organ response (AL-cardiac)	0	N/A (Included AL-cardiac trials prioritized clinical events or safety as primary endpoints).

Abbreviations: 6MWD, six-minute walk distance; CMR, cardiac magnetic resonance imaging; ECV, extracellular volume; GLS, global longitudinal strain; KCCQ, Kansas City Cardiomyopathy Questionnaire; LV, left ventricular; NT-proBNP, N-terminal pro-B-type natriuretic peptide; SAE, serious adverse event; TEAE, treatment-emergent adverse event. Primary outcome domain classification was based on the prespecified primary endpoint listed in the ClinicalTrials.gov registry. Trials reporting multiple primary endpoints were categorized according to the dominant clinical or biological outcome. Hematologic response endpoints were considered distinct from cardiac-specific outcome domains.

**Table 5:** Trial Design and Comparator Characteristics. This table summarizes trial design features, including allocation model, blinding, and comparator type. “Standard of care” comparators refer to background therapy appropriate for the amyloidosis subtype at the time of trial registration. Single-arm studies were defined as interventional trials conducted without a placebo or active comparator.

Study Title	NCT Number	Design Type	Comparator Type
Study of Re-Treatment With ALXN2220 in Patients With ATTR-CM	**NCT07213583**	single-arm/open-label	none/not reported
A Study to Evaluate the Efficacy and Safety of CAEL-101 in Patients With Mayo Stage IIIa AL Amyloidosis (CARES)	**NCT04512235**	parallel-group placebo-controlled	placebo
A Study to Evaluate the Efficacy and Safety of CAEL-101 in Patients With Mayo Stage IIIb AL Amyloidosis (CARES)	**NCT04504825**	parallel-group placebo-controlled	placebo
A Study to Evaluate the Safety and Tolerability of CAEL-101 in Patients With AL Amyloidosis	**NCT04304144**	single-arm/open-label	none/not reported
The Effect of Trimetazidine on Mitochondrial Function and Myocardial Performance in ATTR-CM	**NCT05633563**	crossover/other	placebo
First-in-Human Study of NI006 in Patients With ATTR-CM	**NCT04360434**	parallel-group placebo-controlled	placebo
Doxycycline and Tauroursodeoxycholic Acid (Doxy/TUDCA) in Cardiac ATTR Amyloidosis	**NCT03481972**	parallel-group active-controlled	standard of care (± placebo)
Study of AG10 in Amyloid Cardiomyopathy	**NCT03458130**	parallel-group placebo-controlled	placebo
ION-682884 in Patients With TTR Amyloid Cardiomyopathy	**NCT04843020**	single-arm/open-label	none/not reported
CLEOPATTRA Coramitug Study in ATTR-CM	**NCT07207811**	parallel-group placebo-controlled	placebo
EPIC-ATTR: Eplontersen in Chinese Subjects With ATTR-CM	**NCT06194825**	parallel-group placebo-controlled	placebo
Study of ALXN2220 Versus Placebo in Adults With ATTR-CM	**NCT06183931**	parallel-group placebo-controlled	placebo
A Study to Assess the Safety and Efficacy Of Tafamidis In Chinese Participants With ATTR-CM	**NCT04814186**	single-arm/open-label	none/not reported
A Phase 3 Study of ALXN2060 in Japanese Participants With Symptomatic ATTR-CM	**NCT04622046**	single-arm/open-label	none/not reported
CARDIO-TTRansform: Eplontersen in Participants With ATTR-CM	**NCT04136171**	parallel-group placebo-controlled	placebo
BCD With or Without Doxycycline in Mayo Stage II-III AL	**NCT03401372**	parallel-group active-controlled	standard of care
SGLT2 Inhibitors in Transthyretin Amyloid Cardiomyopathy	**NCT05233163**	single-arm/open-label	none/not reported
A Study of AT-02 in Subjects With Systemic Amyloidosis	**NCT05951049**	single-arm/open-label	none/not reported

Abbreviations: AL, light-chain amyloidosis; ATTR-CM, transthyretin amyloid cardiomyopathy; NCT, National Clinical Trial identifier. Design type and comparator classification were extracted directly from registry-reported study design fields. “Standard of care” comparators refer to background therapy appropriate for the disease subtype at the time of trial registration. Single-arm studies were defined as interventional trials without an active or placebo comparator.

## Data Availability

The original data presented in the study are openly available at ClinicalTrials.gov.

## Abbreviations

The following abbreviations are used in this manuscript:

**Abbreviation**	**Definition**
6MWD	Six-minute walk distance
AL	Light-chain amyloidosis
AL-cardiac	Light-chain amyloidosis with cardiac involvement
ASO	Antisense oligonucleotide
ATTR-CM	Transthyretin amyloid cardiomyopathy
CMR	Cardiac magnetic resonance
ECV	Extracellular volume
GLS	Global longitudinal strain
HF	Heart failure
KCCQ	Kansas City Cardiomyopathy Questionnaire
LV	Left ventricular
mAb	Monoclonal antibody
mRNA	Messenger RNA
NCT	National Clinical Trial identifier
NT-proBNP	N-terminal pro-B-type natriuretic peptide
PCWP	Pulmonary capillary wedge pressure
SAE	Serious adverse event
siRNA	Small interfering RNA
TEAE	Treatment-emergent adverse event
TTR	Transthyretin
